# Development of Autologous Dendritic Cell Vaccine Therapeutics for Canine Mammary Cancer

**DOI:** 10.3390/genes17070794

**Published:** 2026-07-12

**Authors:** Richard Curtis Bird

**Affiliations:** Department of Pathobiology, College of Veterinary Medicine, Scott-Ritchey Research Center, Auburn University Research Initiative in Cancer (AURIC), Auburn University, Auburn, AL 36849, USA; birdric@auburn.edu

**Keywords:** canine, mammary cancer, dendritic cell, cell vaccine, cancer gene defects

## Abstract

Canine mammary tumors have been investigated to determine the causes of malignancy and to promote the development of more effective therapies. The current standard of care, surgical resection where possible, often still results in recurrence of disease. Thus, there is an unmet need for better, more effective therapies that can suppress recurrence in canine patients. Because canine and human mammary cancers, particularly carcinomas and adenocarcinomas, share many similarities in genetic defects, etiology, natural history, and environment, canine mammary cancer cell lines have also been used as effective models of human disease. The genetics and immune response to canine mammary/breast cancers have been investigated to better understand this disease complex and to promote the development of more effective therapies designed to treat individual canine patients. As cancer is a heterogeneous disease, the potential to determine and possibly predict the mechanisms promoting neoplasia would allow the advancement of targeted therapeutic targets/strategies to combat cancer directly. These investigations have led to the development and evaluation of immunotherapies designed to elicit immune recognition of cancer and its suppression, thus improving survival. Hybrid dendritic-cell fusion vaccines and other autologous cancer vaccine formulations have proven effective in suppressing recurrence and extending survival in canine mammary cancer patients following surgical resection. Although current vaccines are somewhat impractical for direct application in veterinary clinics, reported success points the way toward the development of more practical vaccines designed to promote the treatment of canine mammary cancer. They also suggest a possible mechanism whereby removing a tumor from its microenvironment can promote antigenicity by removing local extracellular vesicle-mediated immunosuppression. This review provides a novel perspective on the potential of canine genetics to inform and promote more successful immunotherapies and their value as models of human disease.

## 1. Introduction—Canine Mammary Carcinoma

Canine mammary tumors (CMT) are among the most commonly diagnosed malignancies that occur spontaneously in unspayed female dogs [1,2,3]. Of these tumors, approximately 40–50% are typically malignant and are diagnosed as carcinoma or adenocarcinoma. Dogs are typically more than 6 years old and frequently more than 8 years old when diagnosed, and more frequently represent a geriatric population [3,4,5]. Treatment is typically surgical, involving resection of the tumor followed by chemotherapy if lung metastases are detected by X-ray [3,6]. Dogs diagnosed with carcinoma generally live less than one year; however, even with surgery, they can succumb in months if the disease is extensive. The natural history of mammary carcinoma and adenocarcinoma in dogs closely resembles that of human disease except for the accelerated timeline [7]. Some small differences in frequency between dogs of different breeds or sizes have been reported, suggesting that the majority of tumors are spontaneous and not inherited [6,7,8,9,10,11].

Similar to human breast cancer, canine mammary carcinomas have been separated into luminal A, luminal B, HER2-positive, and triple-negative subtypes based upon gene expression profiles and next-generation transcriptomics [7,12,13]. They typically are categorized as Luminal A or Luminal B phenotypes with expression of estrogen receptor (ER) or progesterone receptor (PR) and expression of HER2 receptor for Luminal B. Significant numbers of ER-/PR- HER2+ tumors, classified as HER2 phenotype, are also encountered [12]. More rarely, triple-negative mammary cancers have been reported, but many of these may also be of an inflammatory phenotype [12,14,15]. This has resulted in a similar subtyping to that used to characterize human breast cancer subtypes. The lack of a standard of care for this disease complex, beyond surgical resection where possible, has focused attention on this unmet medical need [3].

## 2. Canine Mammary Cancer as a Model of Human Breast Cancer

Canine cancers and their development have been employed as models of human cancer due to the similarity in terms of disease, and also because these intermediate-sized patients (between human and mouse) are more comparable to human patients and have intact immune systems [16]. Canine mammary tumors diagnosed in pet animals have been used as exceptional, naturally occurring, and spontaneous models of human disease, as mammary cancers in both species share many features, including environment, histological appearance, tumor genetics, molecular targets, biological behavior, and response to conventional therapies. They also share our lived environment, making the likely causes of spontaneous disease similar as well.

Several labs have developed CMT cell lines as models of human breast cancer because they represent important models of human disease [17,18,19]. These lines take advantage of the tendency for canine mammary tumors to progress at an accelerated timeline, compared to human breast cancer, making them facile patient models that can be evaluated in a shorter timeframe, promoting rapid development and evaluation of new therapeutic strategies [20,21,22,23,24,25].

## 3. Genetic Defects Associated with Canine Mammary Cancer

There has been a concerted search for the gene defects responsible for the development of human cancer since the beginning of modern molecular biology in the 1970s. Canine genetics followed closely as more genetic information on both species became available in the 1990s, and with the publication of the canine genome in 2004 [1,21,26].

There has been steady progress in searching for and identifying the key gene defects promoting mammary cancers in dogs, focusing on known and commonly mutated oncogenes and tumor suppressor genes. These investigations have focused on a group of CMT cell lines originally derived as primary cultures from canine mammary carcinomas [17]. These included CMT12, CMT27, and CMT28 (originally CMT2, CMT4, and CMT5, respectively) and, in some cases, other CMT cell lines [17]. Cells were grown from liberated cancer cells and isolated carcinoma cell cultures established using standard culture techniques and selection in culture or, more recently, high-speed cell sorting [27,28]. Critically for comparison, such analyses also included isolation of a primary population of normal canine mammary epithelial cells derived directly from normal canine mammary gland and subjected to high-speed cell sorting [28]. Assessments of first Northern blots, and then rtPCR assays and Western blots, where functional canine-specific antibodies existed, were used to assess gene candidates and their expression. While these cell lines have been shown to be quite stable, there is always room for caution in comparing cells grown in culture, compared with biopsies directly from tumors. However, heterogeneity of tumor material and contamination with stromal cells also complicates the use of direct biopsies.

This analysis revealed that the most frequent defects encountered were regional deletions, and the gene locus most frequently affected encoded the tumor suppressor protein INK4A/p16 (CDKN2A). Such deletions frequently involved other adjacent INK4 gene family members depending on the size of the deletion [18,29]. Defects in both copies of the INK4A/ARF/INK4B (CDKN2A/B) locus were identified by transcript (cDNA) sequencing [26,27,29,30]. Defects encoding all three members of this cyclin-dependent kinase inhibitor and tumor suppressor gene family (p16/INK4A, p14ARF, and p15/INK4B) were identified [27]. Others have also identified key cell markers for canine mammary gland tumors, and these also reflect similarities to human breast cancers, including some genes associated with hereditary breast cancer [7,31,32,33]. These results confirmed our hypothesis that both canine and human breast cancers share the same key tumor-suppressor defects, strongly suggesting they are important promoters of the cancer phenotype [30].

The CDKN2A locus is surprisingly complex and encodes two distinct proteins from the same locus but employing two different/alternative first exons [19]. Because p16 and p14ARF (Alternative Reading Frame) have different first exons and are then read out of frame, they encode completely different proteins that must coevolve from the same sequence. The complexity arises because the functions of these two proteins both apply the brakes to cell proliferation, but are focused on different protein targets. This occurs because INK4A/p16 functions primarily to suppress CDK4/6 coupled to cyclin D, which phosphorylates the Rb protein, liberating S phase transcription factors (E2F) and thus promoting cell proliferation [17]. INK4A/p16 suppresses this function, allowing continued Rb activity and exit from the cell cycle in G1 phase. When INK4A/p16 is defective, cell proliferation continues without a G1/S phase exit.

In contrast, the alternative open reading frame encoded by the p14ARF protein binds and sequesters MDM2 protein, which functions as an E3 kinase, normally ubiquitinating the p53 tumor suppressor protein and resulting in p53 destruction in the proteasome [19]. Defects in p14ARF result in constitutive p53 destruction and a failure of the apoptotic induction pathway through failure to stabilize p53. The result of defects in the single CDKN2A locus is a failure of both the Rb and p53 pathways and, thus, the failure of both of the key functional brakes on cell proliferation. Because the CDKN2A gene has been found to encode key dysfunctional mutations in many canine cancers, it is, as a consequence, a key oncogenic target [18].

Impact on the p14/ARF-MDM2 pathway has also been implicated directly through detection of p53 tumor suppressor expression defects [34]. Apart from these more canonical breast cancer-associated genes, frequent defects have also been detected in p53 expression, linking these defects to p14/ARF defects [12]. As a consequence, and because these defects are similar to spontaneous human breast cancers, canine mammary tumors have been suggested as promising models for the development of new anti-breast cancer therapeutic strategies [35]. Knowledge of the details of p53 mutations has been rendered more important by recent research suggesting that at least some of the common mutations in p53 can be functionally reversed employing chemical reactivators that have the potential to rescue p53 mutations [36].

Canine mammary cancers have also been shown to be promoted by defects in the expression of hormone receptor genes encoding the estrogen and progesterone receptors, whose expression is strongly correlated with the canine Luminal A and Luminal B mammary cancer phenotypes [12]. This also includes the HER2 receptor (c-*erb*B-2), expressed in canine mammary cancers that are HER2 receptor (c-*erb*B-2) positive (estrogen and progesterone receptor negative) in HER2 phenotype mammary cancers. Activation of expression of other members of the *erb*B gene family, including the epidermal growth factor receptor (c-*erb*B-1), c-*erb*B-3, and/or c-*erb*B-4 genes, can also enhance risk [12].

## 4. Development of Canine Cancer Vaccine Therapeutics

Recent evidence strongly supports the targeting of cancer-specific antigens and cancer neoantigens when developing novel anticancer vaccines [37,38]. Promising results in human and canine patients have been reported despite a long history of limitations as a result of failed attempts to identify and target prominent tumor-expressed antigens. Such antigens often appear to be the result of tactical strategies employed by tumors that deploy prominent antigens that function as molecular decoys to help evade immune surveillance [39]. Spontaneous immune recognition of tumor cells by the patient’s immune system has been observed, although it is not common, reliable, or frequently ineffective in defending the patient from cancer. Immune evasion by tumors is also frequent and often accompanied by loss of expression of MHC class I and MHC class II molecules. Such changes suggest that effective natural antigen presentation within the tumor microenvironment may be defective, possibly through PD-1 binding [40,41]. A different strategy is needed to enhance patient immune defenses.

Armed with detailed knowledge of the genetic defects frequently encountered in canine mammary cancer, investigations of the potential for these tumors to induce an immune reaction capable of systemic targeting of mammary carcinoma cells and rendering an effective response have been attempted. The original premise postulated that canine mammary cancer patients remained sufficiently immunocompetent to mount a killing immune response if they were appropriately presented with tumor cell antigens and were driven to antigen presentation.

The first proof-of-concept report provided indications that this was indeed possible. A canine dendritic cell-like cell line derived from a ten-year-old male Golden Retriever with malignant histiocytosis was employed. DH82 cells were selected as they had been shown to express DC-like characteristics and have been thought to represent an early lineage antigen-presenting cell (APC) in the dendritic cell (DC) lineage [42]. DH82 cells were subsequently shown to express several additional authentic DC surface proteins, including CD40, CD205, and CD209, by RT-PCR analysis, as well as MHC II and CD11c surface antigens, with canine-specific antibodies and flow cytometry [43]. These characteristics suggested that DH82 cells could be promising candidates for fusion with CMT12 or CMT28 cells to create a hybrid-cell fusion that was the basis for a proof-of-concept mammary cancer vaccine that had the potential to force antigen presentation, although they were not autologous patient-derived cells [43].

Vaccination of laboratory Beagles with hybrid-cell fusions accompanied by unmethylated CpG-encoding oligonucleotide immune adjuvants enhanced interferon-gamma expression in sorted CD8+ and CD4+ cells, consistent with a cytotoxic T-cell (CTL) response. Additionally, cell-mediated immune assays (CTL responses) identified significant reactions against both matched and unmatched/unrelated CMT cells [43]. These results strongly suggested that hybrid-cell fusion vaccine constructs could elicit an immune response to mammary cancer antigens that were common to both unrelated cell lines, providing proof of concept for the development of a more generalized cellular vaccination strategy against canine mammary cancer.

Further development of this successful canine mammary tumor therapeutic vaccine was reported, in which the DC-like partner was substituted with authentic autologous dendritic cells isolated directly from canine blood by high-speed fluorescence-activated cell sorting [39]. The newly configured hybrid-cell fusion between autologous canine dendritic cells and unmatched canine mammary tumor cells (CMT28) was designed to promote patient immunity by encouraging authentic antigen-presenting cell recognition and presentation, perhaps through cross-presentation of antigens [44]. Three injections of this construct, with CpG oligonucleotide adjuvants, were effective in eliciting a measurable immune response in laboratory Beagles, including both cytotoxic T-cell lysis and antibody responses. This data confirmed the antigenicity and safety of the hybrid-cell fusion vaccine construct and the potential to successfully vaccinate canine cancer patients therapeutically.

A canine clinical trial was established where patient animals, who had been initially diagnosed with mammary cancer, were treated by surgical resection and had been assessed to have no evidence of metastatic spread to the lungs [45]. All patients were over 8 years old at the time of diagnosis and were geriatric. Each patient animal was vaccinated three times, similar to laboratory Beagles. An additional treatment of Gemcitabine was administered the day prior to each vaccination to potentially suppress regulatory T-cell activity [44,45]. Each patient dog was monitored for physical signs of distress or off-target effects. None were noted in the patient cohort, beyond a mild, transient fever in some animals. Each patient was followed with return visits for one year and then by phone contact for the life of the animal.

**Figure 1:**
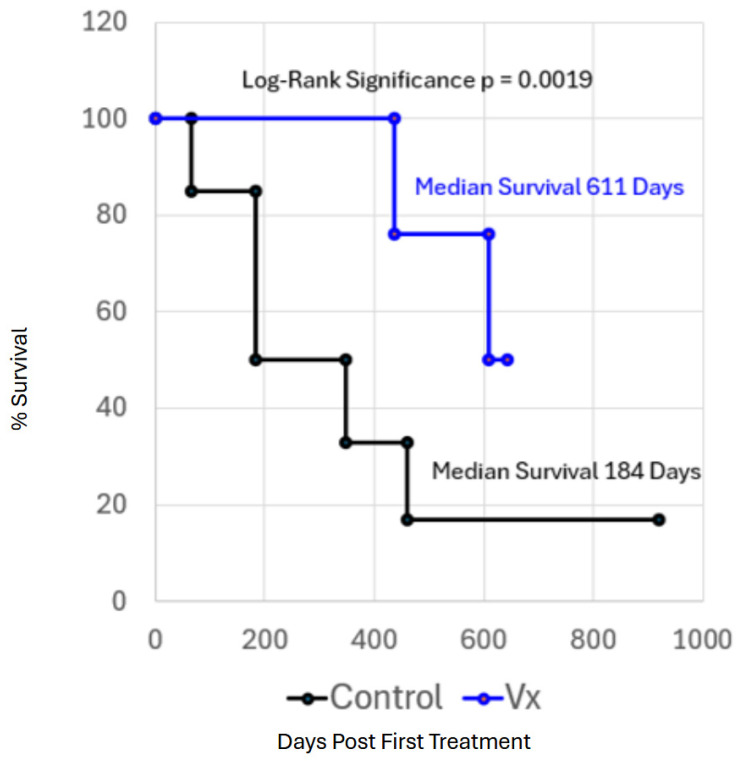
Kaplan–Meier plot of survival after surgical resection alone (black line) or after surgical resection followed by 3 treatments with Gemcitabine and the autologous canine mammary cancer hybrid dendritic cell-fusion vaccine (blue line). Each patient animal was followed with multiple in-person rechecks for one year, followed by telephone checks for the life of the patient or until contact was lost (redrawn from [45]). Calculation of the Log-Rank estimate of significance was *p* = 0.0019. Median survival of the control population was 184 days, while the median survival of the vaccinated population was 611 days. None of the vaccinated patients, whose cause of death was confirmed, died of mammary cancer, and only one patient had a detectable recurrence. Half of these geriatric vaccinated canine mammary cancer patients survived beyond the end of the clinical trial.

In each case, the patient animals survived an average of 3.3-fold longer (median survival of 611 days) than control patient animals (median survival of 184 days) subjected only to surgical resection (Figure 1). Vaccinated animals also appeared to have an enhanced quality of life. Vaccinated animals were followed until death from other causes or loss of contact. No vaccinated animals died as a result of mammary cancer recurrence. In only one case was a detectable recurrence of mammary cancer noted, but this did not cause the death of the patient. As a consequence, the clinical trial was considered a success, as all of the patient dogs were geriatric and outlived the average dog lifespan of 12 years [45].

Some caveats were also evident. Due to the limited nature of the trial, it was not possible to differentiate between the effects of the vaccine and the immune-modifying treatments. Additionally, the trial was limited in the number of patient animals treated. Lastly, the observation of off-target effects, though transient and not requiring treatment, would need to be more thoroughly explored. Such limitations should be addressed in a larger, more thorough clinical trial.

At present, this strategy is not practical for application in the clinic due to cost and the need for advanced cell-sorting technology. However, the development of the technical capability required to identify antigens recognized by vaccinated patients’ immune systems, so that effective generalizable mammary cancer vaccines can be identified, is required. The vaccine could then be reformulated using a peptide- or mRNA-based direct-injection or dendritic-cell-loading strategy [39]. This approach would dramatically reduce the cost and complexity of the hybrid-dendritic cell fusion vaccine and open the possibility for use by veterinary practitioners. No other examples of dendritic cell-based vaccines have been reported in canine patients, although many examples have been reported for human dendritic cells and vaccines, and several promising antigens have been evaluated in canine mammary cancer, as well as reviewed in [6,46].

Other laboratories have also investigated the potential of individual antigens, or, in some cases, tumor neoantigens, for stimulation of canine patients’ immune recognition as an effective defense against cancer development. Although none of these strategies has been evaluated in a canine clinical trial utilizing mammary cancer patients, there has been an evaluation of immunogenicity, and other cancer types have been successfully treated. Expression of the melanoma-associated antigen gene (MAGE) family of surface antigens is associated with a variety of cancers, including mammary cancers [47]. And MAGE antigens are suspected of having potential as key targets in cancer immunotherapy, although canine MAGEs have only been evaluated in mouse models. Gonadotrophin-releasing hormone-based vaccines have also been evaluated in rats, dogs, and Macaca and found to be effective candidates for immunotherapy [48]. Peptides from both of these antigens were successfully presented on MHC molecules.

Canine patients diagnosed with inflammatory mammary cancer have been evaluated for tumor shrinkage as an indication of immune response to in situ injection/vaccination of the neoadjuvant cowpea mosaic virus [49]. This virus was introduced as a novel therapy against canine inflammatory mammary cancer and successfully induced tumor shrinkage in these patients. Immune recognition appeared to be mediated through neutrophil activity and resulted in improved survival of the patients.

These results confirm our hypothesis that environmental context is very important in cancer vaccine development and that the local tumor microenvironment is central to patient immunosuppression guided by the tumor and the microRNA-containing extracellular vesicles it sheds to manipulate the immune response [6,38,50,51,52]. Taking tumor tissue out of this context appears to provide the patient’s immune system with a fresh look at the tumor antigens, possibly promoting immune recognition and successful vaccination. Recently, tissue-intrinsic metabolic activity has been suggested as a possible influence on conditioning anti-tumor immunity in the tumor microenvironment as well as a source of variability in the immune response [53]. Additionally, PD-1 suppression strategies are being developed for canine patients [54,55], and Mammaglobin-A, MMP-7, and mucin-1 have also been evaluated as potential anti-mammary cancer vaccine antigens [6,56,57,58].

Recent work by Suckow and colleagues has reported similar results in several species of domestic animals, including dogs [53,59,60,61,62]. This new work, because it is a different approach utilizing excised patient tumor cells and a biological adjuvant, provides important independent support for this hypothesis. They have demonstrated successful cancer vaccination in canine patients against a variety of tumors using this approach.

## 5. Conclusions

Spontaneous canine mammary cancers and cell lines derived from them have provided an important asset in the development of immune-intact models in which to explore the genetic defects underlying disease. The potential applications for employing immunotherapies to treat canine mammary cancers are promising, although the costs, cell-sorting technology, and human capital required are still beyond the means of practicing veterinarians. However, the potential for using such approaches to evaluate the immunotherapeutic activity of new antigens does provide a new opportunity for discovering and testing new antigens directly in canine patients that are more broadly applicable. The use of individualized mRNA-encoded antigens to immunize dendritic cells in human triple-negative breast cancer patients also provides a new strategy for antigen introduction, expression, and presentation [63]. Because it has a proven track record as a vaccine strategy and is easier to construct, mRNA or protein loading of antigen-presenting cells has the potential to promote the discovery of new and effective canine mammary cancer vaccines that are practical for clinical application [32].

## Data Availability

No new data were created or analyzed in this study.

## References

[1] Ahern T.E., Bird R.C., Church Bird A.E., Wolfe L.G. (1996). Expression of the oncogene c-erbB-2 in canine mammary cancers and tumor-derived cell lines. Am. J. Vet. Res..

[2] Baba A.I., Câtoi C. (2007). Mammary Gland Tumors. Comparative Oncology.

[3] Vail D.M., Thamm D.H., Liptak J.M. (2020). Withrow and MacEwen’s Small Animal Clinical Oncology.

[4] Rupasinghe R., Díaz Cao J.M., Kent M., Lejeune A.T., Rebhun R.B., Martínez-L’opez B. (2026). Descriptive epidemiology of canine and feline cancer in California, United States from 2000 to 2019. Vet. J..

[5] Schwartz S.M., Urfer S.R., White M., Megquier K., Shrager S., Ruple A., The Dog Aging Project Consortium (2022). Lifetime prevalence of malignant and benign tumours in companion dogs: Cross-sectional analysis of Dog Aging Project baseline survey. Vet. Comp. Oncol..

[6] Nosalova N., Huniadi M., Horňáková L., Valenčáková A., Horňák S., Nagoos K., Vozar J., Cizkova D. (2024). Canine Mammary Tumors: Classification, Biomarkers, Traditional and Personalized Therapies. Int. J. Mol. Sci..

[7] Madalina Gherman L., Tomuleasa D., Cismaru A., Nutu A., Berindan-Neagoe I. (2024). Exploring the contrasts: In-depth analysis of human and canine mammary tumors—Discoveries at the frontier. Med. Pharm. Rep..

[8] Carvalho P.T., Niza-Ribeiro J., Amorim I., Queiroga F., Severo M., Ribeiro A.I., Pinello K. (2023). Comparative Epidemiological Study of Breast Cancer in Humans and Canine Mammary Tumors: Insights From Portugal. Front. Vet. Sci..

[9] Dobson J.M. (2013). Breed Predispositions to Cancer in Pedigree Dogs. ISRN Vet. Sci..

[10] Munson L., Moresco A. (2007). Comparative Pathology of Mammary Gland Cancers in Domestic and Wild Animals. Breast. Dis..

[11] Vazquez E., Lipovka Y., Cervantes-Arias A., Garibay-Escobar A., Haby M.M., Queiroga F.L., Velazquez C. (2023). Canine Mammary Cancer: State of the Art and Future Perspectives. Animals.

[12] Lutful Kabir F.M., DeInnocentes P., Agarwal P., Mill C.P., Riese D.J., Bird R.C. (2017). Estrogen Receptor-α, Progesterone Receptor and c-erbB/HER-Family Receptor mRNA Detection and Phenotype Analysis in Spontaneous Canine Models of Breast Cancer. J. Vet. Sci..

[13] Amini P., Nassiri S., Ettlin J., Malbon A., Markkanen E. (2019). Next-generation RNA sequencing of FFPE subsections reveals highly conserved stromal reprogramming between canine and human mammary carcinoma. Dis. Models Mech..

[14] Kim N.H., Lim H.Y., Im K.S., Kim J.H., Sur J.H. (2013). Identification of triple-negative and basal-like canine mammary carcinomas using four basal markers. J. Comp. Pathol..

[15] Burrai G.P., Tanca A., De Miglio M.R., Abbondio M., Pisanu S., Polinas M., Pirino S., Mohammed S.I., Uzzau S., Addis M.F. (2015). Investigation of HER2 expression in canine mammary tumors by antibody-based, transcriptomic and mass spectrometry analysis: Is the dog a suitable animal model for human breast cancer?. Tumour Biol..

[16] Nance R.L., Sajib A.M., Smith B.F. (2022). Canine Models of Human Cancer: Bridging the Gap to Improve Precision Medicine. Prog. Mol. Biol. Transl. Sci..

[17] Wolfe L.G., Smith B.B., Toivio-Kinnucan M.A., Sartin E.A., Kwapien R.P., Henderson R.A., Barnes S. (1986). Biologic properties of cell lines derived from canine mammary carcinomas. J. Natl. Cancer Inst..

[18] Bird R.C., Smith B.F. (2025). Comparative Genetics of Canine and Human Cancers. Vet. Sci..

[19] Lutful Kabir F.M., DeInnocentes P., Bird R.C. (2015). Altered miRNA Expression Profiles Identify miR-141 as the Regulator of INK4A (p16 and p14ARF) Tumor Suppressor Genes in Canine Breast Cancer Models. J. Cell. Biochem..

[20] Paoloni M., Khanna C. (2008). Translation of New Cancer Treatments from Pet Dogs to Humans. Nat. Rev. Cancer.

[21] Ostrander E.A., Wayne R.K. (2005). The canine genome. Genome Res..

[22] Kohn M.H., Murphy W.J., Ostrander E.A., Wayne R.K. (2006). Genomics and conservation genetics. Trends Ecol. Evol..

[23] Meadows J.R.S., Kidd J.M., Wang G.D., Parker H.G., Schall P.Z., Bianchi M., Christmas M.J., Bougiouri K., Buckley R.M., Hitte C. (2023). Genome sequencing of 2000 canids by the Dog10K consortium advances the understanding of demography, genome function and architecture. Genome Biol..

[24] Gherman L.M., Chiroi P., Nuţu A., Bica C., Berindan-Neagoe I. (2024). Profiling canine mammary tumors: A potential model for studying human breast cancer. Vet. J..

[25] Oliveira-Lopes A.F., Götze M.M., Lopes-Neto B.E., Guerreiro D.D., Bustamante-Filho I.C., Moura A.A. (2024). Molecular and Pathobiology of Canine Mammary Tumour: Defining a Translational Model for Human Breast Cancer. Vet. Comp. Oncol..

[26] Lutful Kabir F.M., Agarwal P., Deinnocentes P., Zaman J., Bird A.C., Bird R.C. (2013). Novel frameshift mutation in the p16/INK4A tumor suppressor gene in canine breast cancer alters expression from the p16/INK4A/p14ARF locus. J. Cell. Biochem..

[27] Lutful Kabir F.M., DeInnocentes P., Church Bird A., Bird R.C. (2021). Frequent genetic defects in the p16/INK4A tumor suppressor in canine cell models of breast cancer and melanoma. In Vitro Cell. Dev. Biol.-Anim..

[28] DeInnocentes P., Agarwal P., Bird R.C. (2009). Phenotype-rescue of cyclin-dependent kinase inhibitor p16/INK4A defects in a spontaneous canine cell model of breast cancer. J. Cell. Biochem..

[29] Bird R.C., Yoshida K. (2009). Defects in Genes Regulating the Cell Cycle in Spontaneous Canine Models of Cancer. Trends in Cell Cycle Research.

[30] Lutful Kabir F.M., Alvarez C.E., Bird R.C. (2015). Canine Mammary Carcinomas: A Comparative Analysis of Altered Gene Expression. Vet. Sci..

[31] Klopfleisch R., Lenze D., Hummel M., Gruber A.D. (2010). Metastatic canine mammary carcinomas can be identified by a gene expression profile that partly overlaps with human breast cancer profiles. BMC Cancer.

[32] Yang N.-Y., Zheng H.-H., Yu C., Ye Y., Du C.-T., Xie G.H. (2023). Research progress of good markers for canine mammary carcinoma. Mol. Biol. Rep..

[33] Deckwirth V., Hundi S., Hytönen M.K., Hannula S., Ellonen P., Björkenheim P., Sukura A. (2024). Differential somatic coding variant landscapes between laser microdissected luminal epithelial cells from canine mammary invasive ductal solid carcinoma and comedocarcinoma. BMC Cancer.

[34] DeInnocentes P., Li L.X., Sanchez R.L., Bird R.C. (2006). Expression and sequence of canine SIRT2 and p53 genes in canine mammary tumour cells—Effects on downstream targets Wip1 and p21/Cip1. Vet. Comp. Oncol..

[35] Bhutta Z.A., Choi K.-C. (2025). Canine mammary tumors as a promising adjunct preclinical model for human breast cancer research: Similarities, opportunities, and challenges. Arch. Pharm. Res..

[36] Chen S., Shepard H.M., Lu M. (2026). Reactivating p53 mutants selectively in patients. Cancer Cell.

[37] Sahin U., Schmidt M., Derhovanessian E., Cortini A., Vogler I., Omokoko T., Godehardt E., Attig S., Newrzela S., Grützner J. (2026). Individualized mRNA vaccines evoke durable T cell immunity in adjuvant TNBC. Nature.

[38] Zhang S., Mo S., Huang W., Zhong D., Shenxia Xie S., Aiqun Liu A., Mo F., Xianing X., Liu H., Li Y. (2026). Dendritic cell vaccines: Current research progress, challenges, and opportunities. Genes Dis..

[39] Rutten V.P., Misdorp W., Gauthier A., Estrada M., Mialot J.P., Parodi A.L., Rutteman G.R., Weyer K. (1990). Immunological aspects of mammary tumors in dogs and cats: A survey including own studies and pertinent literature. Vet. Immunol. Immunopathol..

[40] Murgia C., Pritchard J.K., Kim S.Y., Fassati A., Weiss R.A. (2006). Clonal origin and evolution of a transmissible cancer. Cell.

[41] Dolan B.P., Gibbs K.D., Ostrand-Rosenberg S. (2006). Tumor-specific CD4− T Cells Are Activated by “Cross-Dressed” dendritic cells presenting peptide-MHC Class II complexes acquired from cell-based cancer vaccines. J. Immunol..

[42] Wellman M.L., Krakowka S., Jacobs R.M., Kociba G.J. (1988). A macrophage-monocyte cell line from a dog with malignant histiocytosis. In Vitro Cell. Dev. Biol..

[43] Bird R.C., DeInnocentes P., Lenz S., Thacker E.E., Curiel D.T., Smith B.F. (2008). An allogeneic hybrid-cell fusion vaccine against canine mammary cancer. Vet. Immunol. Immunopathol..

[44] Bird R.C., DeInnocentes P., Church Bird A.E., van Ginkel F.W., Lindquist J., Smith B.F. (2011). An autologous dendritic cell canine mammary tumor hybrid-cell fusion vaccine. Cancer Immunol. Immunother..

[45] Bird R.C., DeInnocentes P., Church Bird A.E., Lutful Kabir F.M., Smith A., Smith B.F. (2019). Autologous Hybrid Cell Fusion Vaccine in a Spontaneous Intermediate Model of Breast Carcinoma. J. Vet. Sci..

[46] Qian D., Li J., Huang M., Cui Q., Liu X., Sun K. (2023). Dendritic cell vaccines in breast cancer: Immune modulation and immunotherapy. Biomed. Pharmacother..

[47] Srisawat W., Koonyosying P., Muenthaisong A., Sangkakam K., Varinrak T., Sthitmatee N. (2025). Preliminary Exploration of MAGE-B1, -B4, -B5, and -B10 mRNA Expression in Canine Mammary Tumors in Dogs. Animals.

[48] Junco J.A., Basalto R., Fuentes F., Bover E., Reyes O., Pimentel E., Calzada L., Castro M.D., Arteaga N., López Y. (2008). Gonadotrophin releasing hormone-based vaccine, an effective candidate for prostate cancer and other hormone-sensitive neoplasms. Adv. Exp. Med. Biol..

[49] Alonso-Miguel D., Valdivia G., Guerrera D., Perez-Alenza M.D., Pantelyushin S., Alonso-Diez A., Beiss V., Fiering S., Steinmetz N.F., Suarez-Redondo M. (2022). Neoadjuvant in situ vaccination with cowpea mosaic virus as a novel therapy against canine inflammatory mammary cancer. J. Immunother. Cancer.

[50] Fish E.J., Irizarry K.J., DeInnocentes P., Ellis C., Prasad N., Moss A.G., Bird R.C. (2018). Malignant Canine Mammary Epithelial Cells Shed Exosomes Containing Differentially Expressed MicroRNA That Regulate Oncogenic Networks. BMC Cancer.

[51] Fish E.J., Martinez Romero E.G., DeInnocentes P., Kohler J., Smith A.N., Prasad N., Bird R.C. (2020). Circulating MicroRNA as Biomarkers of Canine Mammary Carcinoma. J. Vet. Intern. Med..

[52] Markkanen E. (2019). Know Thy Model: Charting Molecular Homology in Stromal Reprogramming Between Canine and Human MammaryTumors. Front. Cell Dev. Biol..

[53] van den Berg N.I., Elphick M., Mulder K., Bouricha O., Sadeghi-Alavijeh O., Fu X., Turajlic S. (2026). Immunometabolic gatekeeping: How tissue metabolism conditions tumor immunity. Cancer Cell.

[54] Higgins T.A., Patton D.J., Shimko-Lofano I.M., Eller T.L., Molinari R., Sandey M., Ismail A., Smith B.F., Agarwal P. (2024). The Development and Characterization of a Next-Generation Oncolytic Virus Armed with an Anti-PD-1 sdAb for Osteosarcoma Treatment In Vitro. Cells.

[55] Mei C., Liu Y., Liu Z., Zhi Y., Jian Z., Lyu X., Wang H. (2024). Dysregulated Signaling Pathways in Canine Mammary Tumor and Human Triple Negative Breast Cancer: Advances and Potential Therapeutic Targets. Int. J. Mol. Sci..

[56] Martinez-Romero G., DeInnocentes P., Koehler J.W., Moss A.G., Judd R.L., Bird R.C. (2026). Mammaglobin-A mRNA and Protein Expression Profiles in Canine Mammary Cancer Cell Lines and Tissues.

[57] Martinez-Romero G., DeInnocentes P., Koehler J.W., Moss A.G., Judd R.L., Bird R.C. (2026). Evaluation of Autologous Mammaglobin-A Loaded Dendritic Cells as a Potent Cancer Immunotherapy.

[58] Yadav P.K., Gupta S.K., Kumar S., Ghosh M., Yadav B.S., Kumar D., Kumar A., Saini M., Kataria M. (2020). IL-18 immunoadjuvanted xenogeneic canine MMP-7 DNA vaccine overcomes immune tolerance and supresses the growth of murine mammary tumor. Int. Immunopharmacol..

[59] Lucroy M.D., Clauson R.M., Suckow M.A., El-Tayyeb F., Kalinauskas A. (2020). Evaluation of an autologous cancer vaccine for the treatment of metastatic canine hemangiosarcoma: A preliminary study. BMC Vet. Res..

[60] Lucroy M.D., Kugler A.M., El-Tayyeb F., Clauson R.M., Kalinauskas A.E., Suckow M.A. (2022). Field safety experience with an autologous cancer vaccine in tumor-bearing cats: A retrospective study of 117 cases (2015–2020). J. Feline Med. Surg..

[61] Chelsea B., Greenberg C.B., Javsicas L.H., Clauson R.M., Suckow M.A., Kalinauskas A.E., Lucroy M.D. (2022). Field Safety Experience With an Autologous Cancer Vaccine in 41 Horses: A Retrospective Study (2019–2021). J. Equine Vet. Sci..

[62] Suckow M.A., Hiles M.C. (2023). Use of Conditioned Extracellular Matrix as a Tissue-engineered Tumor Matrisome for Prostate Cancer and Melanoma Immunotherapy. Anticancer Res..

[63] Jo S., Li L., Thakur C., Telfer K.A., Sultan H., Ohara R.A., He M., Nam G., Chen J., Ou F. (2026). mRNA vaccines engage unconventional pathways in CD8^+^ T cell priming. Nature.

